# Foodomics as a Tool for Evaluating Food Authenticity and Safety from Field to Table: A Review

**DOI:** 10.3390/foods14010015

**Published:** 2024-12-25

**Authors:** Shuchen Zhang, Jianan Chen, Fanhui Gao, Wentao Su, Tiejing Li, Yuxiao Wang

**Affiliations:** 1 Dalian Jinshiwan Laboratory, Dalian 116034, China; z090675@163.com; 2 Department of Food Science, College of Light Industry, Liaoning University, Shenyang 110031, China; chenjianan@lnu.edu.cn (J.C.); litiejing@lnu.edu.cn (T.L.); 3 College of Environmental and Safety Engineering, Shenyang University of Chemical Technology, Shenyang 110142, China; gaofh@syuct.edu.cn; 4 State Key Laboratory of Marine Food Processing and Safety Control, Dalian Polytechnic University, Dalian 116034, China; suwt@dlpu.edu.cn; 5 State Key Laboratory of Food Science and Resources, Nanchang University, Nanchang 330047, China

**Keywords:** foodomics, food authenticity, multi-omics strategies, food safety

## Abstract

The globalization of the food industry chain and the increasing complexity of the food supply chain present significant challenges for food authenticity and raw material processing. Food authenticity identification now extends beyond mere adulteration recognition to include quality evaluation, label compliance, traceability determination, and other quality-related aspects. Consequently, the development of high-throughput, accurate, and rapid analytical techniques is essential to meet these diversified needs. Foodomics, an innovative technology emerging from advancements in food science, enables both a qualitative judgment and a quantitative analysis of food authenticity and safety. This review also addresses crucial aspects of fully processing food, such as verifying the origin, processing techniques, label authenticity, and detecting adulterants, by summarizing the omics technologies of proteomics, lipidomics, flavoromics, metabolomics, genomics, and their analytical methodologies, recent developments, and limitations. Additionally, we analyze the advantages and application prospects of multi-omics strategies. This review offers a comprehensive perspective on the food chain, food safety, and food processing from field to table through omics approaches, thereby promoting the stable and sustained development of the food industry.

## 1. Introduction

In recent years, food fraud and adulteration have emerged as global issues, leading to significant discussions about food authentication and traceability, which are closely linked to food safety and quality. It is widely recognized that food fraud is associated with the food trade, driven by economic motives including the intentional addition or substitution of food ingredients, tampering with records, and mislabeling [1]. Surveys indicate that, as consumption levels continue to rise, consumers are increasingly concerned about discerning the authenticity of food through internal and external cues such as origin information, production technology, and organic certifications [2]. Indeed, food authentication has long been a vital area of research in food science. Consequently, detecting food fraud and adulteration, verifying food authenticity, and ensuring food safety and nutrition are essential.

Food authenticity, one of the three major attributes of food, encompasses the undeniable quality, origin, and declaration of food products [3]. In recent years, numerous global incidents involving food authenticity have come to light, such as the substitution of expensive or rare varieties with similar yet cheaper ones. Additionally, there have been instances of deliberately false or misleading declarations regarding the geographical origin [4], production processes, and types of special ingredients used in foods. On the other hand, food adulteration is often accompanied by food safety issues, so food safety, as a food attribute, is also facing challenges around the world. The World Health Organization has repeatedly emphasized that food safety is crucial in multiple links such as food sources, production, processing, and sales. The widespread issue of food fraud and adulteration across the entire food system has both created new challenges and opened up opportunities for the advancement of food authenticity analysis and detection technologies. As methods of food fraud grow increasingly sophisticated, there is a pressing global demand for enhanced food authentication methods. Traditional analytical methods, which are costly and time-consuming, and which require a high level of expertise and professionalism by operators [5], are no longer adequate, thus highlighting the urgent need for a diversified approach to food authentication.

Omics technology is considered a good tool for ensuring food safety and analyzing food authenticity. Therefore, a new discipline, foodomics, has emerged in recent years, combining omics technology with biostatistics, chemometrics, and bioinformatics [6]. Assessments in foodomics have enhanced food quality, enabled traceability, and analyzed the biological activity of food, thus advancing food science research. With ongoing innovations in food science and technology, foodomics has demonstrated its value in elucidating the complex composition and characteristics of food. For instance, metabolomics can be employed to qualitatively and quantitatively analyze metabolites that reflect the nutrition, flavor, and functionality of fermented foods [7]. By detecting spoilage microbial contamination, food pathogens, toxins, and chemical contaminants (pesticides, heavy metals, and veterinary drugs), metabolomics has become a fast, convenient, and efficient tool to ensure food safety [8]. In addition, as a potential separation tool to help separate components in complex samples and obtain good resolution, two-dimensional liquid chromatography (2DLC) is very attractive in food testing. It can separate and detect pesticides, antibiotics, allergens, and naturally dangerous substances (aflatoxins and pyrrolizidine alkaloids), helping to strictly control food safety in the production and processing of food products [9]. Other areas, such as proteomics, flavoromics, genomics, transcriptomics, and lipidomics, are extensively used across various fields of food science to explore key nutrients, evaluate bioactive molecules, and identify and quantify the functional value of foods.

Although targeted and non-targeted methods have been instrumental in identifying and quantifying foods and food ingredients, the development of multi-omics strategies has proven more effective in addressing complex biological processes. For example, proteomics, genomics, and metabolomics have surmounted the limitations of single-omics analysis in understanding the intricate mechanisms of microbial interactions in fermented foods. These strategies have improved fermentation conditions, enhanced the quality and safety of fermented foods, and increased the levels of bioactive metabolites [10]. Similarly, proteomics and genomics approaches have been employed to quantitatively assess the risk of pathogenic microorganisms in dairy products, ensuring food safety [11]. However, the application of multi-omics strategies faces challenges, such as data heterogeneity, which poses significant difficulties in managing and analyzing data across multiple omics platforms, particularly in proteomics and metabolomics. Additionally, the availability of sufficient funding and personnel remains a critical factor [12].

**Figure 1:**
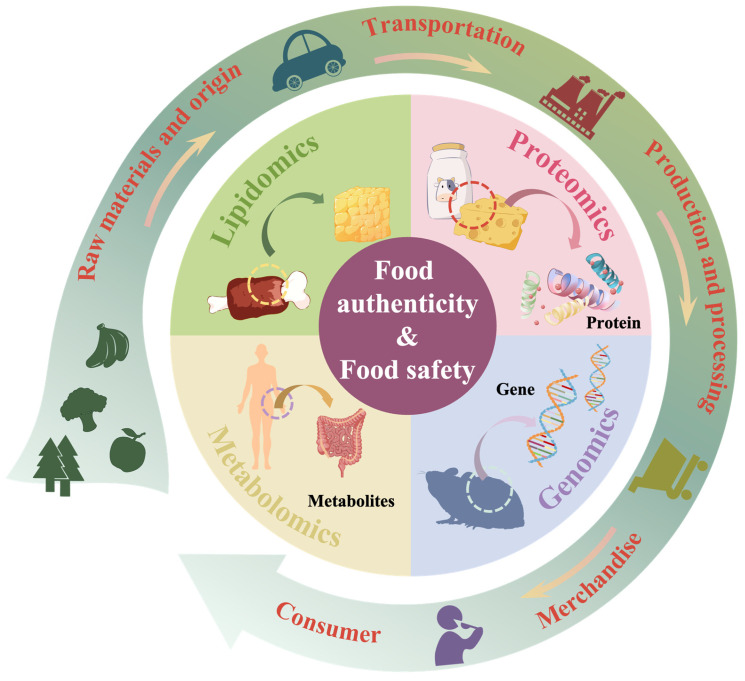
Foodomics is a multi-faceted strategy utilized to analyze both food authenticity and safety.

In this overview, we briefly review foodomics technologies and multi-omics strategies and analyze their recent advancements in the realm of food authenticity. Our discussion encompasses the analysis of food authenticity and safety across various fields, including proteomics, transcriptomics, metabolomics, flavoromics, lipidomics, and genomics (Figure 1). Additionally, we evaluate the advantages and prospects of multi-omics strategies. We aim to employ foodomics to glean meaningful insights into food authenticity from field to table, foster technological innovation in food science, and support the stable growth of the food industry.

## 2. Genomics

Genomics is the study of nucleotide sequence analysis, gene location, genome mapping, and gene function analysis of all genes of a species. DNA is particularly suited for analyzing deeply processed food products and potential contaminants, owing to its stability [13]. Genomics harnesses this capability to thoroughly analyze DNA structure and function. Notably, the precise amplification of DNA fragments through polymerase chain reaction (PCR) technology has become a cornerstone of DNA-based food authentication methods [14], which has profoundly influenced food genomics. A prominent example is the analysis of the olive oil genome; DNA analysis has facilitated the tracing of its geographical origin [15]. Furthermore, Weck et al. employed a real-time PCR assay to detect DNA from *Cannabis sativa*. This innovative method not only accurately identified traces of *Cannabis sativa* but also exposed its adulteration in food products [16]. In the pursuit of food authenticity, genomics frequently plays a crucial role in identifying DNA biomarkers for more precise and substantiated evaluations.

### 2.1. Food Authenticity and Characterization Techniques Based on Genomics Approaches

#### 2.1.1. Meat Products

**Figure 2:**
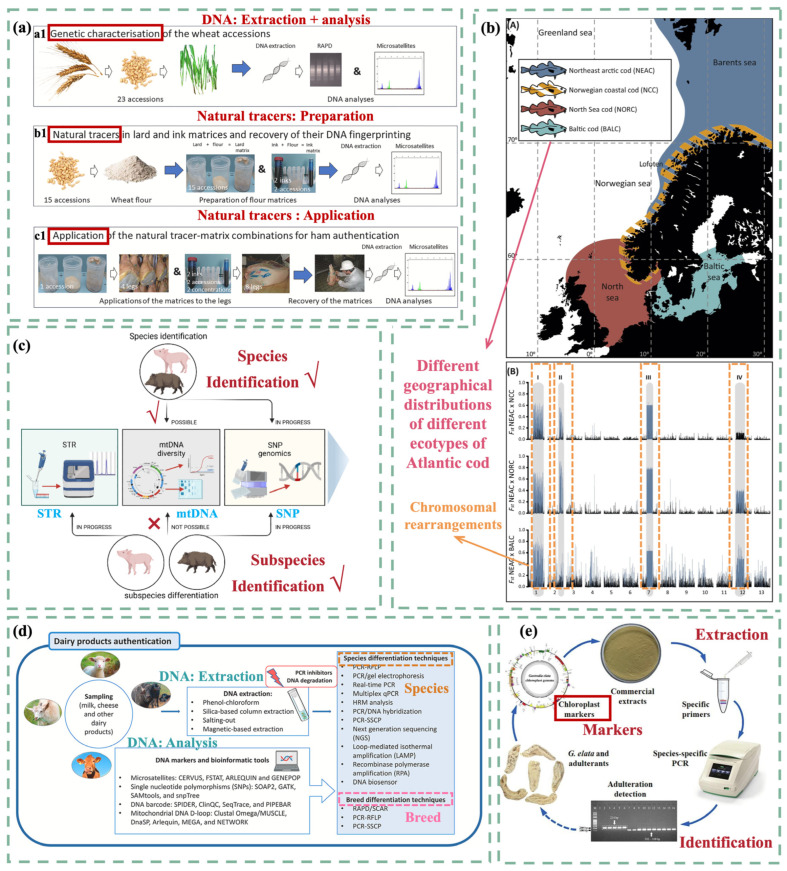
(**a**) Workflow for DNA analysis using natural tracers [18]. (**b**) Geographically undifferentiated but ecologically differentiated seafood populations have generated adaptive variation in only a few genomic regions [24]. (**c**) Method to distinguish domestic pigs from wild boars [19]. (**d**) DNA-based testing method for dairy product authentication [30]. (**e**) Workflow for detecting *Gastrodia elata* Blume adulteration via a specific PCR assay targeting chloroplasts [31].

Meat products are notably prone to fraud, often bearing inaccurate labels regarding species, origin, and brand. Consequently, DNA-based analysis methods are extensively employed to monitor meat products derived from animals. Current technologies enable the identification of species-specific DNA sequences, facilitating the distinction between different species [17]. For instance, in the production of dry-cured ham, wheat or rice may serve as natural biotracers embedded within a lard matrix to prevent drying, and they are included in food-grade inks for labeling. DNA analysis of these biotracers (Figure 2a) allows for the verification of hams from various batches and production years [18]. Despite high genetic similarities, it was difficult to distinguish wild boars from domestic pigs, and finding suitable biomarkers remains a challenge (Figure 2c) [19]. Nevertheless, differential genes present in mitochondrial and nuclear DNA, such as those determining coat color, are anticipated to serve as reliable biomarkers [20].

#### 2.1.2. Seafood

In the realm of fish and seafood, DNA-based technologies typically involve DNA extraction and PCR amplification, with species identification based on analyzed PCR fragments [21]. In 2016, a rapid method for extracting DNA from seafood was introduced, facilitating species identification irrespective of processing and storage conditions [22]. Another research team explored the diversity of five mitochondrial genes in shrimp and developed a PCR method using gene-specific conserved primers to identify various shrimp species [23]. Moreover, genomics has been utilized to track marine products through processing, replacing traditional phenotypic methods. This genomic approach is highly reproducible and accurate, ensuring the verification of species authenticity and labeling information (Figure 2b) [24]. By confirming the authenticity of seafood products, genomics not only promotes the sustainable development of fisheries but also helps stabilize marine ecosystems.

#### 2.1.3. Olive Oil

High-value oils, such as olive oil, particularly those from protected regions, are vulnerable to food fraud [25], including adulteration with cheaper vegetable oils or mislabeling [26]. Traditionally, the authenticity and geographic origin of olive oil have been assessed using metabolite content. While metabolites can confirm the geographical origin, they fall short of accurately assessing the variety and composition of the olive oil [27]. In response, researchers have explored DNA analysis to determine the composition and origin of olive oil. However, DNA extracted from olive oil is prone to degradation, and contaminants such as polysaccharides and polyphenols in the extract may inhibit the PCR reaction [28]. To overcome these limitations, droplet digital PCR (ddPCR) has been proposed, showing significant advantages [29]. As technology advances, improvements in DNA extraction, molecular markers, and analysis platforms continuously enhance the potential of DNA fingerprinting technology in the unambiguous identification of high-value olive oils [27]. Furthermore, during DNA analysis, it is crucial to avoid false-negative results from potential inhibitor contamination and false positive results from inadequate protective measures for the extracted DNA.

### 2.2. Application of Genomics in Food Authentication

In recent years, the development of molecular biology has made it possible for research to no longer rely on the identification of food products based on morphology and metabolic characteristics. The stability of nucleotides makes genomics more suitable for analyzing deeply processed food products and detecting contaminants to ensure food safety. For example, genomics helped characterize fungi involved in food processing and affecting food safety and infer potential functions [32].

#### 2.2.1. Food Traceability

Food traceability enhances food safety and consumer confidence by ensuring that food can be traced through all stages—picking, production, processing, transportation, and distribution [33]. Geographical origin authentication is particularly important for the stable development of the food market [34]. Therefore, developing reliable technologies and platforms for quickly and conveniently detecting the geographical origin of food is of great importance for quality control. DNA barcoding is a tracking method that ensures label authenticity. A DNA-traceable barcode stores various information about the product, swiftly identifying the source and authenticity of the item [35]. On the other hand, compared with protein molecules, DNA molecules have better thermal stability and are resistant to deep processing. For example, DNA identification can be used to trace the origin of collagen peptide products [36].

#### 2.2.2. Species Authentication

The natural world boasts a rich variety of species, particularly in marine environments. When different species coexist in the same area, they may hybridize, posing significant challenges for species identification [37]. Nucleotide sequencing is the most commonly used method for sample identification. For instance, the best close match (BCM) method is capable of identifying both distinct species and their hybrids, as demonstrated in the identification of smooth-shell mussel species [38]. Illustratively, certain PCR technologies related to genomic fingerprinting have been utilized to evaluate chicken in complex meat products, distinguish between different breeds of chickens, and identify chickens with different growth rates [39]. Additionally, fungi are often used to adulterate cordyceps. Specific sequence-characterized amplified region (SCAR) markers have been developed, and real-time PCR has been established to ascertain the species origin, purity, and proportion of adulterants in cordyceps and related materials, thus ensuring food authenticity and ingredient control [40].

#### 2.2.3. Quality Fraud Authentication

The microbiome within food plays a crucial role in determining food quality [41]. Moreover, genomics is frequently employed to classify species and identify microorganisms. The food quality certification discussed here mainly focuses on the microbiome in food. It is widely accepted that investigating the microbiome involves not only the assessment of pathogenic microorganisms but also the overall microbial community within the food, which helps enhance food processing in the context of safety [42]. The NGS-based method has revolutionized research methods concerning food microorganisms. An extended method (gene-targeted NGS analysis) can identify microbial fingerprints for tracking pathogens [43]. Microbial genomics is extensively used in fermented foods to enhance the fermentation function of food-grade microorganisms, resulting in the production of safe and delicious fermented products [44]. For example, in the fermentation process of cocoa beans, researchers used NGS to characterize microbial diversity and found that different volatile compounds would be produced when inoculated with different microorganisms during the fermentation process. For instance, lactic acid bacteria and acetic acid bacteria would produce volatile compounds related to floral, almond, and fruity aromas (such as acetaldehyde, benzaldehyde, etc.) during the fermentation process, *C. metapsilosis* would produce esters related to floral aromas during the fermentation process, *S. cerevisiae* would produce alcohol and be related to floral and sweet aromas, and Lactobacillus would produce compounds related to malt aromas and chocolate aromas (1-butanol-3-methyl and ethanol, 2-nitro) [45].

### 2.3. Limitations of Genomics

Food authentication requires a high purity and yield of DNA for successful PCR analysis. Unfortunately, food processing often leads to protein denaturation and DNA degradation, resulting in the extraction of low-quality DNA [46]. Moreover, during the PCR process, certain compounds in the food or the chemical components used in the extraction process may act as inhibitors of DNA amplification [47]. Therefore, it is necessary to develop new technologies or optimize existing ones to obtain DNA of high purity, quantity, and quality and eliminate potential inhibitors, enabling genomics to more effectively evaluate food authenticity [48]. In addition, the genetic database of food products also needs to be continuously expanded. In the early days, the lack of relevant genetic information led to many people in Japan being poisoned after eating *Pleurocybellaporrigens*, which was regarded as a traditional food but seriously threatened food safety and human health [49].

## 3. Proteomics

Proteomics is recognized as the large-scale analysis of proteins in biological systems, including both quantitative analysis of the proteome and protein profiling. Proteomics is a science that takes the proteome as its research object and analyzes the dynamic changes in protein composition and changes within cells from a holistic perspective. Currently, mass spectrometry (MS)-based proteomics analysis offers improved specificity, accuracy, and sensitivity in the qualitative and quantitative analysis of proteins. For example, MS analysis methods are poised to accurately quantify allergens [50], helping consumers verify the authenticity of food and make suitable, healthy choices. Similarly, due to the huge imbalance between supply and demand, various cheap materials were adulterated into edible birds’ nests. Ma et al. developed a high-throughput method combining shotgun proteomics and scheduled multiple reaction monitoring. It can effectively and quantitatively detect adulterants (including fried porcine skin, swim bladder, egg white, and white fungus) in birds’ nests in the range of 1–80% [51]. In summary, food proteomics has proven to have broad applications in food science, providing suitable biomarkers, aiding in crop development, detecting crop diseases, and ensuring product quality and authenticity.

### 3.1. Food Safety and Authenticity Based on Proteomics

In our daily diets, proteins are major components of many foods, including meat, dairy products, seafood, soy products, eggs, and edible insects [52,53,54]. These protein components can accurately and efficiently reflect the authenticity of food, making it crucial to employ suitable omics methods and technologies for their detection and evaluation. In addition, it is undeniable that proteomics helps understand the structures and functional relations of proteins, which is crucial for detecting potential hazards such as parasites, microorganisms, and allergens in food and strengthening food safety supervision. We have summarized the analytical methods and biomarkers used in proteomics across different foods to evaluate food authenticity.

#### 3.1.1. Meat

With the continuous increase in meat consumption, it is common to find plant protein or other low-priced meats added to meat products. Particularly, lamb, due to its higher price, is more susceptible to mislabeling and food fraud [55]. Past studies have shown rapid development in the use of proteomics for an in-depth exploration of protein changes (such as oxidation [56], hydrolysis [57], and denaturation [58]) that affect meat quality. Researchers have developed a proteomics detection method that combines one-dimensional gel electrophoresis (1DE) with multivariate principal component analysis (PCA), followed by tandem liquid chromatography–mass spectrometry (LC-MS). This method aims to improve chemometrics-mediated gel-enhanced LC-MS (GeLCMS) by using PCA to analyze potential variables in samples. This novel approach enabled the identification of potential protein markers in pork (troponin T, actin cytoplasmic 1, and troponin alpha-1 chain) [59]. Pu et al. utilized the matrix-assisted laser desorption/ionization time-of-flight mass spectrometry (MALDI-TOF-MS) method not only to determine the adulteration rate of beef but also to qualitatively classify both hot-processed meat products and raw meat [60]. Similarly, proteomics is widely used in quality control of pork and pork products [61].

#### 3.1.2. Milk

In the realm of milk and dairy products, proteomics has been employed to verify the presence of dairy and non-dairy proteins to ensure content quality and authenticity [62]. Additionally, proteomics has facilitated the identification of protein biomarkers characteristic of milk from various sources. This capability allows researchers to differentiate between goat milk (GM), buffalo milk, donkey milk (DM), and other milk varieties, thereby assessing milk adulteration. DM and GM are increasingly considered viable substitutes for breast milk due to their high nutritional content and low allergenicity. As such, MALDI-TOF-MS has been utilized to efficiently and accurately detect adulteration in cow’s milk and contamination in DM and GM [63]. Similarly, this method has also been applied to detect adulteration in instances where milk powder is added to fresh cow’s milk [64] or when bovine material is added to milk from other sources [65]. Furthermore, non-targeted UPLC-quadrupole TOF-MS (UPLC-qTOF-MS) has been used to obtain peptide fingerprints. These fingerprints, combined with chemometric analysis, help distinguish authentic milk from samples adulterated with plant proteins [66].

#### 3.1.3. Fish and Shellfish

**Figure 3:**
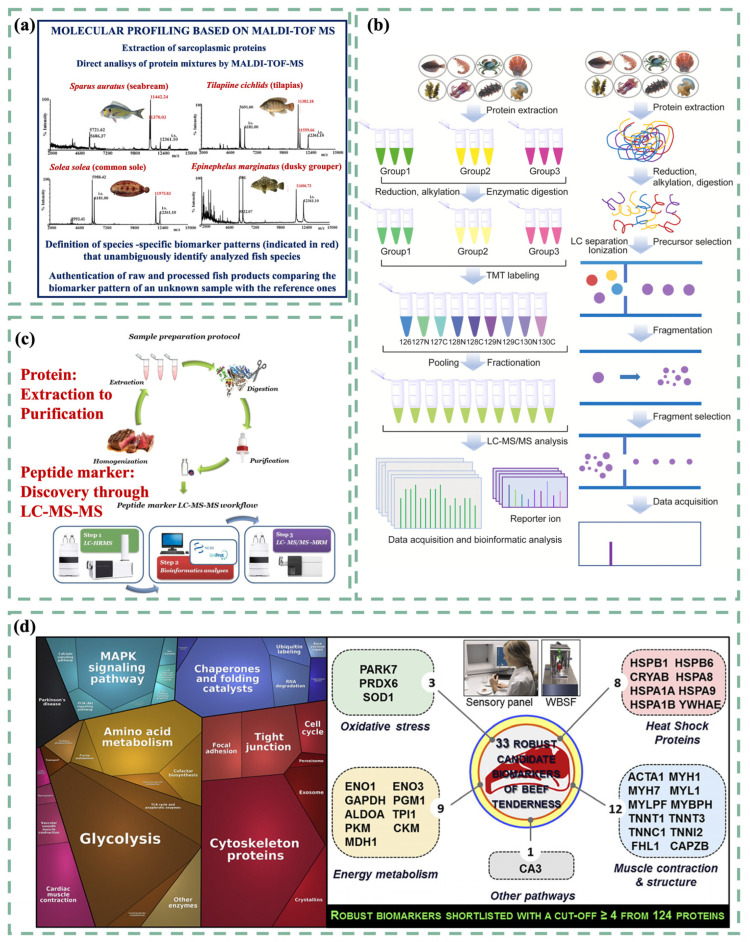
(**a**) Proteomics-based approaches were used to analyze fish-specific biomarkers for species identification [72]. (**b**) Proteomics workflows based on tandem mass tag labeling and multiple reaction monitoring [78]. (**c**) Protein preparation and discovery of peptide markers [79]. (**d**) Potential application of proteomics in the identification of beef tenderness biomarkers [80].

In the global trade arena, seafood products are susceptible to economically motivated food fraud, including mislabeling and substitution. This not only poses food safety risks but also threatens ecosystem protection, as endangered species may be exploited [67]. Unlike genomics, which depends on homology with previously identified proteins to identify “known proteins”, proteomics is crucial for comprehensively describing all proteins present in seafood, such as fish and shellfish [68]. For instance, oyster peptide powder, a highly processed commodity derived from oysters, exhibits multiple biological activities [69]. However, due to its powdered form, it is challenging to distinguish it from other animal or plant-derived peptide powders, leading to a high incidence of adulteration [70]. Proteomics, through the detection of specific peptide biomarkers, has enabled researchers like Li et al. to determine the peptide composition and the original protein sources, thus identifying adulteration in oyster peptide powder [71]. In another study, researchers employed a spectral library matching method to investigate fish species (Figure 3a) [72], using tandem MS-based techniques to overcome the limitations of targeted proteomics, which may arise due to the absence of specific target proteins [73]. These studies demonstrate how proteomics supports the authentication of seafood, aiding consumers in understanding the origins, whether wild-caught or not, of the species involved.

#### 3.1.4. Honey

Traditional methods for assessing the authenticity of honey are time-consuming and laborious, and the range of discrimination is limited. As protein is one of the important components of honey, protein-based omics methods can reveal the adulteration of honey in detail [74]. In 2021, researchers verified the application of qualitative proteomics in exploring New Zealand mānuka honey certification. This highly specific method discovered 12 peptides, including PM1-PM8 and their variants, as potential biomarkers, as well as bee-derived proteins, mainly major royal jelly proteins (MRJPs) [75]. On the other hand, MRJP was the main protein in honey protein, so proteomics detected the authenticity of honey by quantitatively detecting MRJPs as an endogenous marker [76]. However, in current research, the quantitative analysis of honey is more focused on the quantitative analysis of candidate peptides of different MRJPs by calculating the peak areas in the selected reaction monitoring chromatogram [77].

### 3.2. Application of Proteomics in Food Authentication

Proteomics can accurately identify and quantify biomarkers in food products. As an extremely sensitive detection method, proteomics identifies label errors, determines biological sources, and detects samples contaminated with contaminants.

#### 3.2.1. Geographical Origin Authentication

Traceability in animal-derived foods remains a crucial aspect of food authenticity. Currently, alongside stable isotope analysis [81] and multi-element analysis [82], proteomics is also employed to determine the geographical origin of foods. Thus, MS-based proteomics plays a significant role in exploring the authenticity and traceability of “blue foods”. For example, Zhang et al. used the sequential window acquisition of all theoretical fragment ion mass spectra (SWATH-MS), combined with chemometrics, to analyze *Apostichopus japonicus* (*A. japonicus*) from various regions in China. Given that A. japonicus is a precious sea cucumber species, its geographical origin significantly influences its nutritional value and market price, underscoring the importance of authenticating its origin [83]. The research team leveraged SWATH-MS-based proteomics for quantitative analysis to delineate the geographical origins of this product [84].

#### 3.2.2. Species Identification

In the food market, substituting high-value species with inferior ones has been a common form of fraud, especially when the species involved are difficult to distinguish visually. Recently, proteomics has made significant contributions to investigating food authenticity, as demonstrated in the authentication of fish species [85]. A notable example involved researchers using MALDI-TOF-MS to analyze and classify samples of Nile perch and Barramundi. They developed a classifier capable of distinguishing Barramundi from Nile perch. It was also found that other fish species were classified as neither Barramundi nor Nile perch, with both raw and cooked fish samples being correctly identified [86]. This method proved particularly suitable for classifying fish tissue samples, as fish species are closely related, and it is impractical to test the genomes of all species. Similarly, to address the problem of adulteration in Indian water buffalo, researchers developed two-dimensional gel electrophoresis (2DE) and OFFGEL-based proteomics methods to identify mixed meats [87].

#### 3.2.3. Quality Control

Developing more accurate and sensitive methods to identify biomarkers and qualitatively and quantitatively analyze proteomic expression data has become the preferred approach to enhancing food quality control [88]. This approach was clearly illustrated in studies deciphering variations in beef quality. Gagaoua et al. used integrative methods to study proteins related to beef tenderness, aiming to use identified protein biomarkers to quickly verify beef tenderness and better manage beef quality [80]. Additionally, MALDI-TOF-MS was employed to examine the source of cheese product ingredients and enhance understanding of dairy product quality [89]. In seafood, the edible body wall of sea cucumbers contains collagen, a biomacromolecule of great commercial value [90]. Researchers chose proteomics over traditional SDS-PAGE analysis and discovered that the collagen molecules in fibrillar collagen were heteromorphic [91]. This proteomic study of collagen could assist in the improved processing of sea cucumber products.

Targeted proteomics, on the other hand, could accurately and reproducibly quantify the proteins in a sample, which was crucial for studying the allergens in food. MS analysis methods were poised to accurately quantify allergens, including β-PRVBs, fructose bisphosphate aldolase, and creatine kinase in fish [50], helping consumers verify the safety of food and make appropriate and healthy choices.

### 3.3. Limitations of Proteomics

In practical applications, proteomics may face certain limitations. For example, dairy products are sterilized via heat treatment during production, which can induce the Maillard reaction [92]. Techniques such as tandem MS play a crucial role, but currently, they cannot identify all modified molecules in dairy products or elucidate the chemical structures of derivatives [93]. Most of the search engines related to proteomics are still insufficient or wrong for the field of halal food analysis, which may be due to the fact that halal food usually comprises highly processed meat products. This makes the analysis more complicated [94]. Furthermore, when identifying marine fish species, proteomics based on specific peptide tags suffers due to the absence of a reference proteome for the target species, greatly limiting its application. Despite these limitations, proteomics remains a promising method for verifying food authenticity.

## 4. Metabolomics

**Figure 4:**
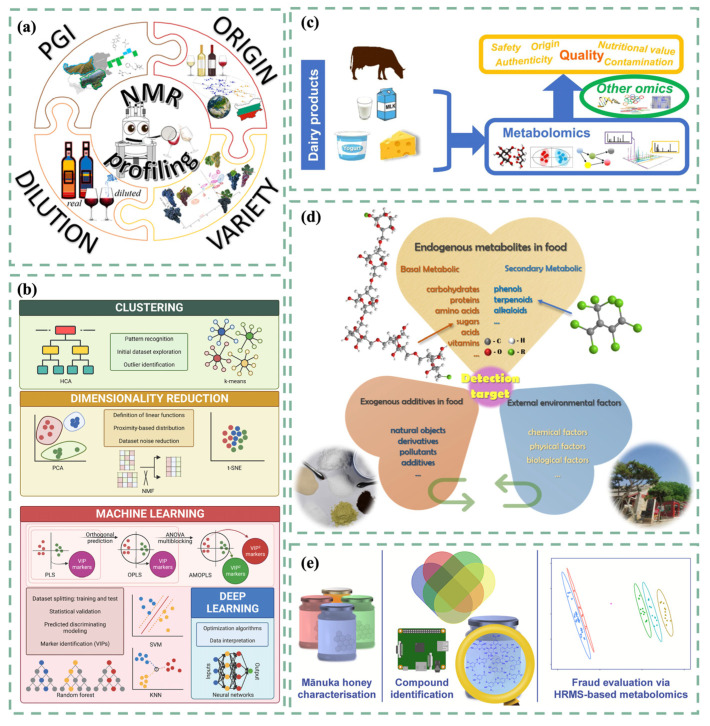
(**a**) NMR was used to authenticate wine [102]. (**b**) Overview of the most widely used unsupervised (**top**) and supervised (**bottom**) chemometric algorithms in food metabolomics [103]. (**c**) Metabolomics was used to evaluate the authenticity of milk and dairy products [104]. (**d**) Food metabolites include endogenous metabolites, exogenous additives, and small molecules in the environment [105]. (**e**) Metabolomics helped identify potential markers of Mānuka honey [106].

**Figure 5:**
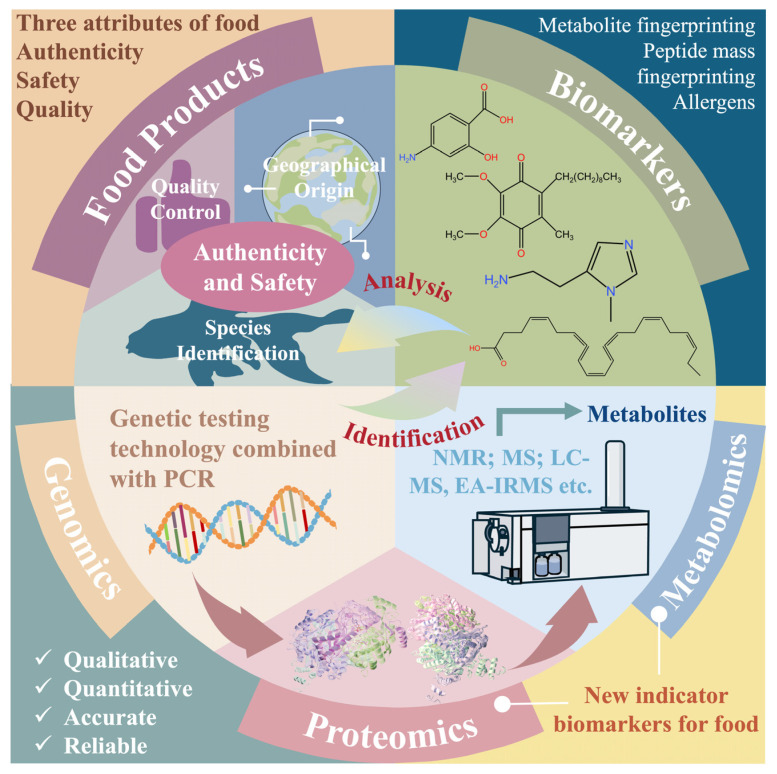
Discovery of biomarkers and their association with food product authenticity through a systems molecular approach.

Metabolomics refers to the study of metabolic pathways in biological systems by observing changes in metabolites in biological systems after being stimulated with external stimuli, and it involves studying the concentrations and interactions of low-molecular-weight metabolites in samples. This is crucial for detecting food authenticity. Generally, metabolomics is categorized into targeted and non-targeted methods. Traditionally, targeted methods were heavily relied upon for identifying food fraud. However, the melamine scandal exposed limitations in targeted detection, highlighting the need for non-targeted methods. For example, the authenticity of high-value items such as saffron has always garnered significant attention due to its high cost. Traditional targeted technologies, like high-performance LC and nuclear magnetic resonance (NMR) spectroscopy, were only capable of detecting specific cases of adulteration, limiting their broader application [95]. Consequently, researchers have been developing non-targeted metabolomics methods that can identify the authenticity of saffron under complex adulteration conditions through the chemical fingerprints of phenolic compounds [96]. Additionally, recent years have seen efforts to integrate metabolic analysis with multivariate statistics to better manage and interpret the data obtained, revealing insights hidden within (Figure 4a) [97]. Moreover, as shown in Figure 5, we summarize the biomarkers found in food that can be used to assess the geographical origin, quality, and species of food products.

### 4.1. Authenticity Evaluation of Dietary Food Ingredients by Metabolomics

Metabolomics, as an emerging technology in recent years, holds significant potential for discovering biomarkers that help assess food quality and authenticity. Through the qualitative and quantitative analysis of metabolites, it is used to distinguish the geographical origin of meat, differentiate between conventional and organic products, and identify the plant origins of honey (Figure 4e) and wine (Figure 4c), among others [98].

#### 4.1.1. High-Value Meats

Given the demand for high-value meats, cheaper meats are often intentionally adulterated into food products. For example, in the current market, beef balls are a popular product; however, some businesses use pork for adulteration, which is difficult to detect. To address this issue, a non-targeted method combining LC resolution MS (LC-HRMS) and PCA has been developed to identify pork content in beef and certify halal food [99]. Similarly, other inexpensive meats, such as donkey and chicken, are also likely to be mixed with higher-value meats. Metabolomics using ^1^H-NMR can distinguish metabolites from different sources and screen out creatine, choline, and carnitine, etc. as metabolites that distinguish chicken from red meat, and lactate, inosine, and pyruvate, etc. as metabolites that distinguish donkey meat from mutton and beef [100]. Additionally, GC-MS-based metabolomics has been employed to differentiate mechanically recycled meat, which is not defined as meat, from hand-deboned meat [101].

#### 4.1.2. Milk and Dairy Products

In 2014, GC-MS was used to distinguish goat milk from cow milk based on their metabolites, and it could detect as little as 5% cow milk in goat milk [107]. As technology has progressed, metabolite markers (D-biotin and the peptide of Thr-Ala-Val) of non-organic milk adulterated in organic milk have been detectable via ultra-performance LC–tandem MS (UPLC-MS/MS) [108]. Additionally, mothers who lacked sufficient breast milk or chose not to breastfeed found that human milk sharing could be a more convenient way to access donor human milk. However, this breast milk was susceptible to adulteration [109]. Earlier DNA and protein-based detection technologies were limited by a lack of targeted probes, low sensitivity, and lengthy processes. By contrast, as shown in Figure 4b, metabolomics offers a rapid quantification method for adulterants. The chemical isotope labeling LC-MS method has proven to be more accurate and comprehensive in detecting breast milk adulteration, capable of identifying adulterants at levels of 5% or higher [110]. Furthermore, metabolomics serves as a powerful tool to thoroughly understand the metabolites in dairy products, thereby analyzing their safety and quality. As early as 2012, high-resolution magic angle spinning NMR (^1^H HRMAS-NMR) was utilized to identify specific metabolites in Mozzarella di Bufala Campana and statistically analyze the cheese’s geographical origin and aging process [111]. Becchi et al. employed a combination of non-targeted metabolomics and chemometrics to assess the authenticity and quality of Parmigiano Reggiano cheese [112].

#### 4.1.3. Wine

Wine is currently the most popular alcoholic beverage worldwide. Its properties are influenced by various factors, including grape growth, origin, climate, soil, storage conditions, and production equipment. Hence, authenticating its origin and production details is essential. Wine adulteration often involves misrepresentation regarding the geographical origin of the grapes or the year of production. As a reproducible, non-destructive, and representative method, ^1^H-NMR is highly suitable for detailed analysis of wine characteristics such as color, production method, origin, and grape variety [102]. The same method was also used to evaluate Bordeaux wines. The results showed that more phenethyl alcohol, gallic acid, and arabinose distinguished Bordeaux wines from other French wines [113].

#### 4.1.4. Tea

Non-targeted metabolic fingerprinting analysis helps understand the relationship between tea composition and variety quality. Based on LC-MS non-targeted metabolomics, researchers screened a variety of differential metabolites from different tea varieties and found that high-quality green tea had higher contents of flavonoid glycosides, flavonoid glycosides, and anthocyanin glycosides. This was crucial for the subsequent variety identification, traceability, and certification of tea [114]. Similarly, Zeng et al. used metabolomics to find that 10 compounds such as benzyl benzoate, heptane, and quercetin-3-O-galactoside could be used to distinguish different grades of *Tieguanyin* [115]. In summary, metabolomics is widely used in the tea industry by focusing on the differences in metabolites in tea of different varieties, growth environments, grades, and types.

#### 4.1.5. Tuna

Metabolomics has been instrumental in verifying the authenticity of fish and seafood products, particularly through species identification. Tuna, being one of the most valuable food items, is frequently subject to fraudulent practices that can be combated through species identification. Recently, Hu et al. reported using GC-Q-TOF/MS and UPLC-Q/Orbitrap/MS to analyze the metabolic characteristics of tuna, demonstrating good predictability of the results [116]. In addition, ^1^H NMR-based metabolomics provided a wealth of metabolite information, including lactate, creatine, carnosine, etc., to distinguish species and sources of wild tuna [117].

#### 4.1.6. Fruit Juice

Juice is one of the most popular drinks in various countries. However, the conflict between supply and demand has been exacerbated by rising costs, making the juice industry vulnerable to adulteration. Some advanced omics technologies have, therefore, been applied to comprehensively evaluate the authenticity of juice. For example, Han et al. used a high-performance liquid chromatography (HPLC) method combined with fluorescence detection to identify and screen oxygenated heterocyclic aglycones in orange and grapefruit juice. It was found that this method could detect 10% mandarin juice adulteration in pure sweet orange juice and 10% pomelo juice adulteration in grapefruit juice, which was also the lowest adulteration [118]. Another typical example was pomegranate juice. Targeted and non-targeted analysis of pomegranate juice metabolites via NMR can be used as a detection method to distinguish adulteration [119]. In this study, the quantitative equal-carbon response HSQC experiment was considered a promising quantitative tool to help quantify key metabolites (glucose, citric acid, alanine, etc.).

### 4.2. Application of Metabolomics in Food Authentication

In the fields of food authenticity and food safety, chromatography combined with mass spectrometry can help detect, quantify, and interpret compounds in complex matrices. Therefore, metabolomics based on technologies such as NMR can simultaneously analyze different or similar mass compounds, elucidate the metabolic pathways and interactions of compounds, and determine metabolic rates [120].

#### 4.2.1. Geographical Identification

The traceability of food and feed has always been a critical concern. In today’s increasingly complex supply chains, metabolomics is particularly suited for studying the origin of products because the metabolome is closely linked to the environment. A prime example is the study of plants; metabolomics can reveal abiotic effects on plant metabolites and serve as a powerful tool for authenticating food in species biogeography [121]. For instance, the metabolome of corn used for feed and starch production has been analyzed using ultra-performance LC combined with MS, and 20 potential biomarkers, such as triglycerides, diglycerides, phosphatidylcholine, and phosphatidylethanolamine, were discovered [122]. Additionally, *Coffea arabica* from Ethiopia, known for its distinctive flavor and aroma, holds significant market value. Metabolomics, closely related to phenotype and significantly influenced by external factors, has great potential to safeguard the uniqueness of this regionally grown coffee, thus ensuring the correct geographical indication [123].

#### 4.2.2. Determination of Species Labels

Consumers have the right to know the species and geographical origin of their food, as well as whether their food, particularly aquatic products, is wild or farmed [124]. For instance, HRMS combined with chemometrics has been utilized to successfully distinguish between shrimp species. This non-targeted metabolomics approach has identified specific markers that quickly verify a product’s identity [125]. Furthermore, sea buckthorn berries, which have various medicinal and edible uses across multiple species and subspecies, have also been studied. Using ^1^H-NMR-based metabolomics, researchers have differentiated three species of sea buckthorn berries and identified potential metabolite markers (L-quebrachitol, organic acids, etc.) [126].

#### 4.2.3. Improvement in Quality

Metabolomics has become extensively used in the food industry, with a particular focus on enhancing food nutrition and quality, which is now a research priority. In this field, researchers often combine mass spectrometry with chromatography techniques to achieve more accurate mass detection. The detection of volatile compounds, as well as primary metabolites, could provide information on quality deterioration, such as the unpleasant smell of tilapia during freezing [127]. Moreover, in one experiment, metabolomics was employed to evaluate various quality aspects of wine, including taste parameters, flavor compounds developed during aging, and microbial metabolism [128].

### 4.3. Limitations of Metabolomics

Although metabolomics has greatly advanced in food authentication, no single technique can comprehensively identify the entire metabolome. Each metabolite is chemically distinct, and each instrument has its strengths and limitations [129]. For example, NMR displays low sensitivity, requires large sample volumes, and relies on small databases [120]. In addition, the identification of metabolites mostly relies on known databases. However, only some databases can realize batch search of MS’, while the search for MS^2^ is still difficult. This will lead to a large number of false positive results. Additionally, the use of molecular network technology in metabolomics introduces challenges such as sample complexity, variability in food ingredients, and the potential for false positives and negatives, all of which can restrict its application [130]. Therefore, selecting the appropriate method for the research sample is crucial for the reliability of metabolomics studies.

## 5. Lipidomics

Given the diversity and complexity of lipids, some of which are present in low concentrations, the emerging field of lipidomics has gained significant attention. Lipidomics involves the analysis of lipids and lipid-like compounds within complex samples. In the context of blue foods, lipidomics aids in species identification, quality and nutritional evaluation, and differentiation between wild and farmed fish [131].

Moreover, lipidomics can be divided into targeted and non-targeted approaches. Targeted lipidomics focuses on specific lipids, particularly those with characteristic fragmentation patterns or those present in low concentrations [132]. In contrast, non-targeted lipidomics provide a broad spectrum of metabolic features, which is useful for discovering new lipid biomarkers. For example, to prevent the adulteration of durum wheat with common wheat in pasta production, researchers have analyzed metabolites from both types of wheat separately. This approach not only identifies markers to distinguish between the two but also detects adulteration levels as low as 3% [133].

### 5.1. Safety Assessment of Edible Health Ingredients via Lipidomics

#### 5.1.1. Lipid Profiles in Tissues

Various types of meat possess unique lipid profiles, which lipidomics can leverage to enhance meat testing. Each species has specific tissues characterized by unique fatty acids that help differentiate between meat types in products [134]. For example, a GC-MS analysis of halal foods showed that beef sausages contained higher levels of palmitic acid, lower levels of myristic acid and oleic acid, and a unique presence of stearic acid compared to pork sausages [135]. Elemental analyzer–isotope ratio mass spectrometry (EA-IRMS) can also differentiate between samples with the same chemical composition. For example, significant differences in carbon isotopes could be used to differentiate animal fats, potentially allowing for halal certification [136]. Similarly, since the carbon isotope ratio in plant samples could be measured, EA-IRMS was also used to determine the authenticity of honey. The difference in carbon isotope ratios between C4 and C3 plant-derived honey could be used to determine whether the honey was adulterated [137]. Additionally, for detecting mislabeling, a lipidomics method that combines machine learning (ML) and rapid evaporation–ionization MS (REIMS), has successfully identified the lipid fingerprint of sliced beef, accurately detecting mislabeled beef 100% of the time [138].

#### 5.1.2. Milk Lipids

Milk lipids are significant nutrients and components of milk, exhibiting multiple biological activities. Lipidomics facilitates component analysis, quality assessment, the identification of geographical origin, and authenticity verification in milk and dairy products [139]. Zhang et al. employed non-targeted lipidomics to study the changes in milk lipids following different heat treatments. The results showed that oxidized lipids (oxidized triglycerides) distinguished the heating conditions of milk, while triglycerides, phosphatidylcholine, diglycerides, etc. distinguished raw milk from pasteurized milk and extended the shelf life of milk [140]. In 2022, UPLC-Q-TOF-MS/MS was utilized to identify and analyze polar lipids in whey [141]. The milk-fat globule membrane, which is beneficial for neurodevelopment and gastrointestinal health, has also been characterized in butter processing by-products using combined lipidomics and proteomics, providing detailed characterizations of the different by-products [142]. These experiments demonstrate that MS-based lipidomics technology can rapidly detect and analyze a wide array of lipid groups in samples.

#### 5.1.3. Nuts

Nuts are rich in nutrients, and they are good dietary supplements. They are added to various processed foods, especially pastries, sauces, and cooking oils. As allergens, food fraud involving nuts can seriously threaten human health. On the other hand, lipids are one of the representative components of nuts. Therefore, lipidomics analyzes, identifies, and quantifies lipids in extracted nuts to evaluate the traceability and authenticity of nuts. For example, Ma et al. used non-targeted lipidomics to study lipid oxidation in peanuts stored in nitrogen-conditioned storage (NS). It was observed that, compared with conventional storage, NS delayed phospholipid metabolism and glycerophospholipid monomers, thereby delaying lipid oxidation in peanuts [143]. In addition, lipid research in plant-based beverages was also a good example. The researchers analyzed triglycerides in plant milk from almonds, soybeans, and coconuts, respectively. The results showed that, in addition to different types of triglyceride molecules, almonds and soybeans also contained unconventional fatty acids [144].

#### 5.1.4. Commercial Fish Species

**Figure 6:**
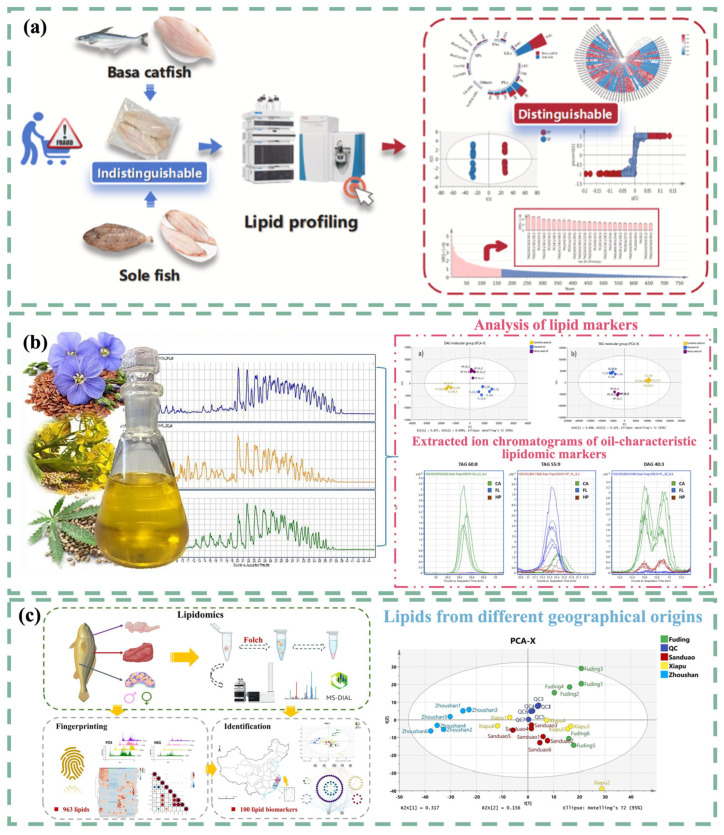
(**a**) Workflow for the analysis of basa catfish and sole fish using UHPLC-QE Orbitrap MS technology [147]. (**b**) Analysis of lipids from three cold-pressed seed oils via LC/Q-TOF MS [149]. (**c**) Mass spectrometry-based lipidomics research process for LYC lipid composition [150].

Many important commercial fish species worldwide are prone to economically motivated food fraud, including the adulteration and mislabeling of tuna, grouper, halibut, etc. To address this, Song et al. proposed a method using an electrometric knife (iKnife), coupled with REIMS, to quickly analyze the lipid profiles of four tuna species and verify their identities [145]. Similarly, Lu et al. employed the same method to authenticate shrimp species in highly processed minced shrimp products [146]. In 2024, the research team developed ultra-high-performance liquid chromatography-QE Orbitrap MS (UHPLC-QE Orbitrap MS) technology for comprehensive statistical analysis of the lipid composition of sole fish and basa catfish (Figure 6a), highlighting the potential of lipidomics in verifying the authenticity of fish products [147]. Chen et al. used non-targeted lipidomics to explore the changes in lipids in fish during fermentation. They found that triglycerides and phosphatidylcholine were the main lipids in fermented fish, and glycerophospholipid metabolism was considered a potential metabolic pathway in the fermentation process [148].

### 5.2. Application of Lipidomics in Food Authentication

Lipidomics helps us extensively study the composition, structure, and relationship of lipids in food products, as well as the impact of changes in lipid composition during food harvesting, processing, transportation, and storage on food products.

#### 5.2.1. Regional Distribution

The quality and composition of some foods are closely linked to their geographical origin, which can lead to significant price variations in the market. Lipidomics plays a crucial role in origin authentication. GC-MS was used to analyze the unsaponifiable fraction (UF) and triglyceride (TAG) spectrum of hazelnuts, and the combination of UF and TAG fingerprinting facilitated rapid and relatively accurate identification of the geographical origins of hazelnuts [151]. A study on the lipid spectrum of large yellow croaker (LYC) revealed that different environmental factors correlate differently with lipids [150], supporting the use of lipidomics based on unique lipid characteristics as a promising strategy for identifying geographical origins.

#### 5.2.2. Species Authenticity

In recent years, with advances in technology, lipidomics-based food inspection methods have enabled the rapid identification of sample identities. Hu et al. used MS-based lipidomics to qualitatively and quantitatively analyze the lipid molecules of three commercial tuna species in order to identify the species [152]. Lipidomics has also made significant contributions to the identification of meat, grains, vegetable varieties, and muscle tissues [153,154,155].

#### 5.2.3. Evaluation of Quality Changes

An increasing number of studies have explored the use of lipidomics to detect changes in food quality. One direct application is the authentication of edible oils. Chromatography combined with mass spectrometry (LC/Q-TOF MS) has been utilized to obtain the lipid profiles of cold-pressed oils and identify the unique lipid characteristics of each oil. These results underscore the potential of lipidomics to distinguish between edible oils and detect adulterants [149]. Additionally, lipidomics has facilitated the discovery of markers related to the quality of goat meat, including tenderness, flavor, meat color, and water retention capacity [156]. Qu et al. employed non-targeted lipidomics to investigate the effects of storage conditions on the lipid metabolism of brown rice, highlighting the role of low-temperature storage in preserving brown rice quality [157].

### 5.3. Limitations of Lipidomics

Lipidomics faces several challenges in practical applications. For instance, the concentration of many lipids is very low, necessitating enrichment steps for subsequent detection. Furthermore, lipids are sensitive to oxidation, and certain specific lipids are also light-sensitive. This instability requires that lipids be freeze-dried and stored away from light during extraction and analysis to prevent degradation. Additionally, the lipidome of most foods has been minimally studied at the molecular level [158]. In summary, researchers need a detailed understanding of the lipidome, must select targeted detection technologies to efficiently and sensitively evaluate lipids, and need to further develop comprehensive databases. Table 1 lists the applications of omics technologies in assessing food authenticity.

**Table 1:** Foodomics technology application in food and product evaluation of food authenticity.

Type of Food	Food Certification Issue	Biomarkers	Omics Technology	Refs
Meat	Halal meat	Myofibrillar proteins	GeLCMS	[59]
	Water buffalo	Species-specific peptide biomarkers	2DE and OFFGEL-based proteomics	[87]
	Beef–pork	Fifteen potential metabolite markers including *cis-5-tetradecenoylcarnitine*, decylubiquinone and 3-methylsulfolene, etc.	LC-HRMS employing Orbitrap mass analyzer and PCA	[99]
Milk and dairy products	DM and GM	Total spectrum in the mass range of 2000–25,000 Da	MALDI-TOF-MS	[63]
	Organic jersey milk	The peptide of Thr-Ala-Val and D-biotin	UPLC-MS/MS	[108]
	Organic yak milk	Trimethylamine *N*-oxide		
	Breast milk	Five metabolites	CIL LC-MS	[110]
	Geographical origin of Mozzarella di Bufala Campana	Isobutylic alcohol, lactic acid, and acetic acid	^1^H HRMAS-NMR	[111]
	Aging of Mozzarella di Bufala Campana	β-galactose, β-lactose, acetic acid, and glycerol		
Seafood	Oyster peptide powder	Signature peptides (GPPGKPGP, SGPAGPR, and GPPGPAGPA)	Nano-LC-MS/MS and LC-MRM-MS/MS	[71]
	Fish species	29 fish species in the North Sea and the scripts	Spectral library tandem mass spectrometry-based method	[72]
	*Apostichopus japonicus*	Actin and actin isoform 2, major yolk protein 1 and 2, vasa-like protein	SWATH-MS combined with chemometrics	[84]
	Nile perch and Barramundi	Fish peptide	MALDI-TOF-MS and a classifier	[86]
	Tuna	Thirty-eight metabolites including 4-Aminosalicylic acid and 3-Methylhistamine, etc.	GC-Q-TOF/MS and UPLC-Q/Orbitrap/MS	[116]
	Tuna	Phospholipids	UPLC-QE Orbitrap MS	[152]
	Frozen/thawed fish and fresh fish	EPA and DHA	LC-HRMS/MS	[159]
Wine	Conventional wine vs. Bulgarian local wine	Most sugars such as galactose and xylitol and alcohols	^1^H-NMR	[102]
	Grape varieties	Most acids such as succinic acid, tartaric acid, lactic acid, etc.		
	Diluted wine	Fructose		
	Geographical origin	Acetoin and coutaric acid, etc.		
Spirits	Mulberry brandy	Ethyl acetate, ethyl hexanoate, ethyl caprylate, ethyl laurate, and ethyl oleate; acetaldehyde, furfural, and vanillin; Propanol, 2,3-Butanediol; Eugenol	Solid-phase microextraction and GC-MS	[160]
Cereals	Durum wheat	Heptadecyl-resorcinol	Non-targeted lipidomics	[133]

## 6. Muti-Omics Strategies for Full Food Processing

In practical applications, the integration of data from multiple sources is critically important for ensuring food safety supervision, enhancing food quality control, advancing food nutrition research, and verifying food authenticity. The use of multiple omics technologies in food research is increasingly common. Considering the impact of post-harvest processing on *Radix Gentianae Macrophyllae*, Sun et al. combined metabolomics and proteomics to study the molecular mechanism. This comprehensive analysis not only included the overall metabolic changes of gentian before and after processing but also supplemented the effects of enzymes in plant tissues on active components [161]. This combination of multiple omics analysis methods not only helped screen suitable post-harvest processing technologies to improve the accumulation of active ingredients but also provided a reference to improve the quality of processed food or pharmaceutical products. Similarly, Ji et al. used non-targeted proteomics and metabolomics to evaluate fingerprint proteins and metabolite markers of mare’s milk (MM) samples after adulteration with bovine milk. The results showed that, in protein analysis, zinc-α-2-glycoprotein, osteopontin, and serotransferrin detected 1% adulteration in pasteurized mare’s milk (PMM), but the adulteration limit was increased to 10% in mare’s milk powder (MMP). In metabolite analysis, N6-Me-adenosine showed an adulteration limit of 1% in PMM, but as low as 0.1% in MMP [162]. Non-targeted metabolomics combined with proteomics has emerged as a powerful approach to detecting adulterants (pork) in fish meat, with myoglobin and β-hemoglobin used as peptide markers to identify pork [163]. Both non-targeted metabolomics and proteomics achieved the determination of adulterants at the lowest concentration level (0.5% *w*/*w*), making the halal certification of fish products more standardized and more reliable. Wen et al. found that genomics can identify the species of deer in different regions, but for the same species in different regions, NMR-based metabolomics could better analyze the metabolites in different regions. Therefore, combining the two omics analysis methods can make the traceability of deer antlers more reliable [164]. By combining metabolomics and lipidomics to respectively reveal the differences in polar and non-polar metabolites between pork and lymph nodes, the authors determined metabolites that had the potential to distinguish them. A combined factor regression equation was established, and it was stipulated that the adulteration of lymph nodes could be determined when the combined factor exceeded 0.75 [165]. Table 2 summarizes the application of multi-omics strategies in food authenticity verification.

The multi-omics approach does not merely combine multiple omics technologies; it enables a more comprehensive analysis by overcoming the limitations inherent in individual omics methods [166]. This approach facilitates a deeper understanding of the regulatory and connective processes among molecules, organisms, and species. It also allows for more accurate, rapid, and convenient food inspections and enables the extraction of more meaningful data and insights.

**Table 2:** Analytical methods for multi-omics strategies in applications.

Food Type	Authenticity Factor	Muti-Omics Strategies	Refs
Milk and dairy products	Mare milk mixed with cow milk	Proteomics–metabolomics	[167]
	Mare milk powder and mare milk mixed with bovine milk	Proteomics–metabolomics	[162]
	Cream	Proteomics–lipidomics	[168]
Meat	Pork adulterated with beef	Metabolomics–lipidomics	[169]
	The quality of pork from different parts	Proteomics–lipidomics	[168]
	Pork with lymph nodes	Metabolomics–lipidomics	[165]
Seafood	Pork-adulterated *Pangasius hypothalamus* meat	Proteomics–metabolomics	[163]
	Tuna adulterated with pork	Proteomics–metabolomics	[170]
Other food	Geographical origins of deer antlers	Genomic–metabolomic	[164]
	Effect of the formation of characteristic volatiles in preserved egg yolk during pickling	Metabolomics–flavoromics	[171]
	Flaxseed varieties	Metabolomics–lipidomics	[172]

## 7. Conclusions and Trends

In recent years, food fraud has become a global issue, affecting not just specific species or regions but also posing significant risks to consumer safety, health, and interests worldwide. Common practices within the global food industry include species fraud, mislabeling, ingredient fraud and adulteration, and the use of substandard concentrations.

Foodomics is a discipline that assesses the composition, nutrition, quality, and authenticity of food. Compared to traditional detection methods, foodomics offers advantages such as speed and convenience in identifying food fraud and food safety control. It enables the analysis of DNA, metabolites, proteins, and lipids in food products using high-throughput techniques. The data obtained are then analyzed to determine factors related to food authenticity, such as geographical origin, species, ingredients, production methods (wild or farmed), and processing techniques. By integrating various omics methods, researchers are progressively elucidating the complex interactions between food and biological systems. Therefore, the detailed technologies and methods of different omics in representative food applications were discussed, as well as their specific applications in the traceability, processing, and production of food products. In addition, the combination of multi-omics technologies, as well as the connection between and comparative advantages of individual omics technologies, is indispensable for exploring the prospect of multi-omics strategies. This helps us systematically analyze the application prospects of food omics technology in food authentication.

Although high-resolution mass spectrometry provides more sensitive data for research with the advancement of mass spectrometry technology, there are still some problems. For example, the number of representative biomarkers is very large, and the existing database is insufficient. Direct analysis in real-time mass spectrometry achieves direct injection, but matrix effects, complex component separation, and the lack of food-related databases are its limitations. These issues will become the focus of future research. Currently, these limitations have been noted, and many researchers are working to expand databases and innovate data-sharing technologies. Studies have begun to invite the sharing of retention time (RT) data in non-targeted metabolomics. This effort may make RT sharing as common as sharing MS/MS data, providing more data for metabolomics identification. More and more similar initiatives have been proposed, and many people believe the quantitative distribution of metabolites and key details of lipidomics analysis should be made available as sharing data.

Therefore, considering these issues, the relevance and application of multiple foodomics technologies in the same study are increasingly explored. Some researchers have also realized that foodomics combined with artificial intelligence achieved more advantages in processing data sets from a wide range of sources. This not only solves the limitations of a single omics group but also allows for a multi-faceted discussion of the problem, thereby gaining a more comprehensive understanding of the authenticity of food, including food quality, traceability, and origin.

In summary, the future of foodomics looks promising for studying bioactive compounds in food. We can anticipate more accurate testing data, standardized methods, and more user-friendly testing systems in the future. The diligent planning and implementation of multi-omics technologies will play a crucial role in advancing food authentication research.

## Data Availability

No new data were created or analyzed in this study. Data sharing does not apply to this article.
